# Antenatal experiences of pregnant women with cardiac conditions: a systematic review and meta-synthesis

**DOI:** 10.1016/j.xagr.2025.100522

**Published:** 2025-05-28

**Authors:** Jasmine X. Kiley, Annabelle Corlett, Emma Mitchell-Sparke, Brittany Jasper, Tabitha Wishlade, Catriona Bhagra, Sara Wetzler, Catherine E. Aiken

**Affiliations:** 1 Department of Obstetrics and Gynaecology, University of Cambridge, Box 223, The Rosie Hospital and NIHR Cambridge Biomedical Research Centre, Cambridge, United Kingdom (Kiley, Mitchell-Sparke, Jasper, Wishlade and Aiken); 2 Department of Clinical Medicine, University of Cambridge, Addenbrooke’s Hospital, Cambridge, United Kingdom (Corlett); 3 Tufts University School of Medicine, Boston, MA (Mitchell-Sparke); 4 Department of Cardiology, Addenbrooke’s and Royal Papworth Hospitals, Cambridge, United Kingdom (Bhagra); 5 Department of History and Philosophy of Science, University of Cambridge, Free School Lane, Cambridge, United Kingdom (Wetzler); 6 Department of Obstetrics, Gynecology, and Reproductive Science, Icahn School of Medicine at Mount Sinai, New York, NY (Wetzler)

**Keywords:** qualitative study, systematic review, cardiac disease, antenatal, high-risk pregnancy, patient-provider relationship, patient experience

## Abstract

**Objective:**

Cardiovascular conditions are the leading cause of maternal mortality in high-income countries. We aim to inform supportive care that addresses the needs of pregnant women with cardiac conditions.

**Data Sources:**

Medline via Ovid, Embase via Ovid, CINAHL via Ebsco, PsychINFO via Ebsco, Scopus, Web of Science Core Collection, and ASSIA via Proquest were searched, database inception-June 2024.

**Study Eligibility Criteria:**

Studies with qualitative components describing experiences of pregnant women with any cardiac condition globally.

**Study Appraisal and Synthesis Methods:**

The Critical Appraisal Skills Programme checklist for qualitative research was employed to perform quality assessment. Inductive coding and thematic analysis were conducted using NVivo software.

**Results:**

Thirteen qualitative studies met the inclusion criteria for meta-synthesis. We identified 3 key themes pertaining to the pregnancy experience of women with cardiac conditions, including patient-provider risk perception matching, importance of communication, and peer influence. Women with established versus new cardiac diagnoses in pregnancy had differences in their experiences. Depending on their own perception of risk, women noted over-medicalization or dismissal by their providers. Our findings also showed that some women sought peer support through online support groups, which either provided a sense of community and hope, or were anxiety-inducing.

**Conclusions:**

It is critical for women and providers to have nuanced and personalized discussions about the risk of cardiac conditions during pregnancy. Experience of pregnancy differs amongst women with new or pre-existing cardiac conditions and is based on women’s appraisal of their pregnancy’s risk level. Reaching an agreement in risk perception is crucial to strengthen the patient-provider relationship and provide a basis for women to feel secure during their pregnancy.

**AJOG Global Reports at a Glance**

**Why was this study conducted?**

This study was conducted to establish a framework for supportive and effective care for women with cardiac conditions experiencing pregnancy.

**Key findings**

Congruence in patient-provider risk perception strongly influences pregnancy experience, particularly how much trust women have in their healthcare team. Communication quality, including whether women feel heard, has a strong impact on the likelihood of patients and providers reaching a consensus on risk perception.

**What does this add to what is known?**

Our contextual framework may improve understanding of the pregnancy experience of women with cardiac conditions by providing context as to how provider communication influences patients. Patient-provider risk perception congruence is a key element of women having a secure and non-stressful pregnancy experience.

## Introduction

Cardiovascular conditions are the leading cause of maternal mortality in high-income countries.^1^ In the United States in 2019, cardiac conditions accounted for up to 33% of maternal mortality,^2^ while in the United Kingdom and Ireland, they caused 13% of deaths during pregnancy between 2020−2022 and remained the largest single etiology of indirect maternal deaths, excluding COVID-19.^3^ As advances in congenital heart disease (CHD) management continue, increasing numbers of women with CHD are reaching childbearing age.^4^ In the US, there was a 25% increase in the number of women becoming pregnant with an existing heart condition between 2003 and 2012.^3^ Furthermore, the incidence of cardiomyopathy, including peripartum cardiomyopathy (PPCM), is also increasing among pregnant women.^3^

Women with cardiac conditions in pregnancy are at risk of adverse outcomes, including maternal and fetal mortality.^5^, ^6^, ^7^ Pregnancy care for these women must be carefully tailored towards reducing these risks; however, there is a paucity of research examining how women experience this close monitoring.^8^ In view of the increasing number of women impacted by cardiac conditions in pregnancy, it is important to provide a framework for understanding these women’s experiences. This will inform the development of care pathways that simultaneously meet women’s needs and optimize their pregnancy outcomes.

A previous systematic review focused on the information needs of women with cardiac conditions considering pregnancy and how coordination of care may be achieved.^8^ Women noted insufficient information availability and disjointed care services, which may be alleviated through shared decision making and communication across the clinical team. We build on these important findings by focusing specifically on how women with new or known cardiac conditions experience prenatal care, and how prenatal pathways can be designed to optimize both psychological and obstetric outcomes. As the number of women affected by cardiac conditions in pregnancy rises, an increasing number of studies have reported their experiences, and thus many of the studies available for inclusion in this review have been published in the last 6–7 years.^9^, ^10^, ^11^, ^12^, ^13^, ^14^

We performed a qualitative systematic review exploring the pregnancy experiences of women with new or existing cardiac conditions who experienced antenatal care, aiming to inform supportive care that addresses the needs of pregnant women with cardiac conditions.

## Methods

### Search strategy

This systematic review is reported according to Prisma guidelines.^15^ The protocol for the review was prospectively registered with the international prospective register of systematic reviews (PROSPERO: CRD42023484456). Seven databases were searched (Medline via Ovid, Embase via Ovid, CINAHL via Ebsco, PsycINFO via EBsco, Scopus, Web of Science Core Collection, and ASSIA via ProQuest). Initially, studies pertaining to any cardiac condition in conjunction with pregnancy were located, and then only those specifically addressing the experiences of pregnant women with cardiac conditions were included (A.1). Manual searches of the reference lists of included studies, Google Scholar, and previous systematic reviews were conducted to complete a comprehensive search. All searches were completed January 5, 2025.

### Selection criteria

All original qualitative or mixed-methods (with qualitative findings reported) studies in peer-reviewed journals that met the inclusion criteria were included. Eligibility criteria for the participant population included any pregnant woman with a cardiac condition, either pre-diagnosed or newly diagnosed during pregnancy. Studies that were not directly focused on women’s experiences of care, studies in languages other than English, and publications other than primary research papers (e.g. review articles) were excluded. Following de-duplication, all articles were screened by 2 reviewers (JK, AC or BJ). Each reviewer independently screened records using Rayyan software, blinded to each other’s inclusion decisions.^16^ A third reviewer was available to resolve conflicts. Full texts of all articles that met inclusion criteria were obtained.

### Assessment of risk of bias

The Critical Appraisal Skills Program toolkit was used to assess all studies. Two reviewers undertook independent assessments (JK & EM), and a third was available to resolve conflicts.

### Data synthesis

We extracted data using NVivo v.15 software to code first-order (participant quotations) data. Second-order data (researcher interpretations, including themes and descriptions of findings) was used to contextualize the data and support the coding process. Two reviewers (JK & AC) verified the extracted data, and disagreements were resolved in discussion with a third reviewer.

Two authors (JK & CA) performed the inductive thematic analysis, developing descriptive analytical themes and a framework that outlined the ways in which risk perception mismatch of patient and provider can influence the experience of women under cardio-obstetric prenatal care.

## Results

### Study selection

Our search yielded 18,052 studies. After removing duplicates, 10,038 unique studies were screened (A.1). We included 13 studies of 388 pregnancies in the final thematic synthesis (Table 1).

**Table 1:** Summary of included primary studies

Study	Country	Diagnosis	Study focus	Qualitative prompts	Data collection	Data analysis	Summary of themes	No. of references
de Wolff, Mie et al. 2018	Denmark	Peripartum cardiomyopathy (n=24)	Experiences of psychological adaptation after having PPCM	Opening question and probe examples provided	Interviews	Thematic analysis	Recovering to a new normal after PPCM, losing trust, silence after chaos, disruption of early mothering, choices made for me and not by me, ability to mobilise inner resources	47
Dekker, Rebecca et al. 2016	International online support groups (My Heart Sisters, A Woman’s Heart, PPCMnet)	Peripartum cardiomyopathy (n=92)	Experiences of women after being diagnosed with PPCM	N/A	Narratives from online support groups	Thematic analysis	Symptom dismissal and misdiagnosis, strong emotional reactions upon learning of diagnosis, concerns about impact of PPCM on future childbearing	19
Donnenwirth, Jo Anne and Hess, Rosanna 2018	United States	Peripartum cardiomyopathy (n=16)	Explore experiences of women living with PPCM and their decisions regarding a subsequent pregnancy (SSP)	Open-ended sample questions provided	Interviews	Constant comparison analysis	Risk of relapse into heart failure impact decisions about future pregnancies, receiving the ultimatum ‘no more children’, weighing the risks of a SSP, making the decision about a SSP, experiencing a SSP	31
Hess, Rosanna et al. 2012	International online support group (MySpace)	Peripartum cardiomyopathy (n=148)	Describe contents of postings made on MySpace by women diagnosed with PPCM	N/A	Narratives from online support groups	Thematic analysis	Discussion of symptomology, exchange of advice, interactions with healthcare providers, uncertainty of future pregnancies, expressions of spirituality, recovery from heart failure	21
Hess, Rosanna et al. 2010	United States	Peripartum cardiomyopathy (n=12)	Determine benefits of participation in the online support group for PPCM	Open ended and Likert style questions provided	Descriptive survey	Thematic analysis	Benefits of being in group, how support group helps me, what I learned from others in group, what I gain as part of group	38
Hutchens et al. 2022	Australia	Tetralogy of Fallot (n=2), bicuspid aortic valve (n=1), mitral valve prolapse (n=1), hypertrophic cardiomyopathy (n=5), arrhythmogenic right ventricular dysplasia (n=1), long QT syndrome (n=2), bicuspid aortic valve and patent ductus arteriosus (n=1), left ventricular noncompaction cardiomyopathy (n=1), idiopathic cardiomyopathy (n=1), pregnancy-associated spontaneous coronary artery dissection (n=10), patent foramen ovale (n-1), peripartum cardiomyopathy (n=1)	Explore and understand the healthcare experiences of women with cardiac disease in pregnancy and postpartum	Interview guide not specified	Interviews	Inductive reflexive thematic analysis	Dismissed: struggling to be heard; Too little, too unclear: in search of information; Winging it: research, education, and guidelines; Fragments: care co-ordination and continuity; Making do: fitting into services designed for others	42
Hutchens et al. 2022	Australia	Congenital heart disease (n=4), genetic heart disease (n=8), acquired heart disease (n=12), congenital and genetic heart disease (n=1)	Correct the lack of visibility and information on the experiences of women with cardiac disease in pregnancy and the first year postpartum	Opening question provided, interview guide not specified	Interviews	Interpretive inductive thematic analysis	Ground zero: index events and their emotional and psychological impact; Self-perception, identity and worthiness; 1 the road alone: isolation and connection	63
Mayer, Felicity et al. 2018	United Kingdom	Cardiac disease (arrhythmic or structural) (n=15)	Describe composition and processes of multidisciplinary care between maternity and cardiac services and women’s experiences of delivering/receiving care within these models	Interview topic guide specified	Retrospective case note audit (n=42 women), interviews (n=15 women, n=11 clinicians)	Thematic analysis	Model of integrated care, influence of clinicians’ specialist interest in pregnancy and cardiac conditions on model of care, clarity of acquired vs. congenital pathways, congenital conditions, acquired conditions, midwifery involvement, normality	
Ngu, Kylie et al. 2014	Australia	Low risk congenital heart disease (n=10), high risk CHD (n=10)	Motivations and perceptions of women with low vs. high risk CHD following successful pregnancy	Interview guide not specified	Questionnaire (n=20), interviews (n=20)	Thematic analysis	Influence of external expectations placed on women to have children, influence of existing relationships, woman’s goals and desired experiences, innate biological urges coupled with understanding of reproductive changes with age, termination of pregnancy not considered viable option	23
Ngu, Kylie et al. 2014	Australia	Congenital heart disease (n=20)	Assess perceptions of women with CHD regarding severity of cardiac abnormality and its implication in pregnancy	Interview guide specified in an additional file	Interviews	Thematic analysis	Reliance on clinicians’ care and dependency on successful outcome based on improved medical and surgical management, distorted understanding of their condition, past experiences of (successfully) living with heart defect and its (non-impact) on their quality of life, adaptation to living with CHD	24
Patel, Harshida et al. 2016	Sweden	Peripartum cardiomyopathy (n=19)	Explore and describe women’s experiences of symptoms in peripartum cardiomyopathy	Opening question and probe examples provided	Interviews	Qualitative content analysis	Being caught in a spider web, invasion of the body by experienced symptoms and feeling of helplessness	41
Patel, Harshida et al. 2016	Sweden	Peripartum cardiomyopathy (n=19)	Explore women’s experiences of health care while being diagnosed with PPCM	Open-ended and probe sample questions provided	Interviews	Qualitative content analysis	Exacerbated suffering, not being cared about, not being cared for, not feeling secure	40
Vasconselos Amorim, Thaís et al. 2018	Brazil	Rheumatic heart disease (n=6), Ischemic heart disease (n=2), Congenital heart disease (n=1), Arrhythmia (n=2), Mitral insufficiency (n=4), Dilated cardiomyopathy (n=2)	Understand meaning of pregnancy for women with heart diseases	Guiding questions provided	Interviews	Hermeneutical analysis	Knowing the risk and planning and ignoring how to avoid it and getting surprised when finding out pregnancy, telling how they felt physically and emotionally during pregnancy, feeling safe due to the prenatal follow-up routine	20

### Study characteristics

Studies were conducted in 6 countries (Australia, Brazil, Denmark, United Kingdom, United States, Sweden), encompassing 4 continents. Studies also included data from internationally accessible online platforms. At least 18 distinct cardiac diagnoses were reported across the studies (Table 1). The research methods used in the included studies included either contemporaneous or retrospective in-depth interviews (n=10), surveys (n=1), and analysis of narratives from online support groups (OSGs) (n=2). The analytic methods utilized in the studies included thematic analysis, constant comparison analysis, hermeneutical analysis, and content analysis.

### Risk of bias of included studies

The overall quality of the included studies was good, with all papers meeting the Critical Appraisal Skills Program (CASP) 18 evaluation on the majority of key points (A.2). We identified 6 studies with an unclear relationship between researcher and participants, 3 studies with ambiguous recruitment strategies, and 1 study with unclear research design and ethics and that lacked rigorous data analysis and a clear statement of findings.

### Synthesis of results

A key theme running throughout the included studies centered around perception of risk, which was a powerful influence on how women experienced pregnancy. An important interplay was apparent between the woman’s own perception of the risk of her cardiac condition and that of her care-providers. When women perceived that their provider expressed similar levels of vigilance and concern about their cardiac condition as the women felt themselves, they were much more likely to report feelings of safety and trust.

**Figure 1:**
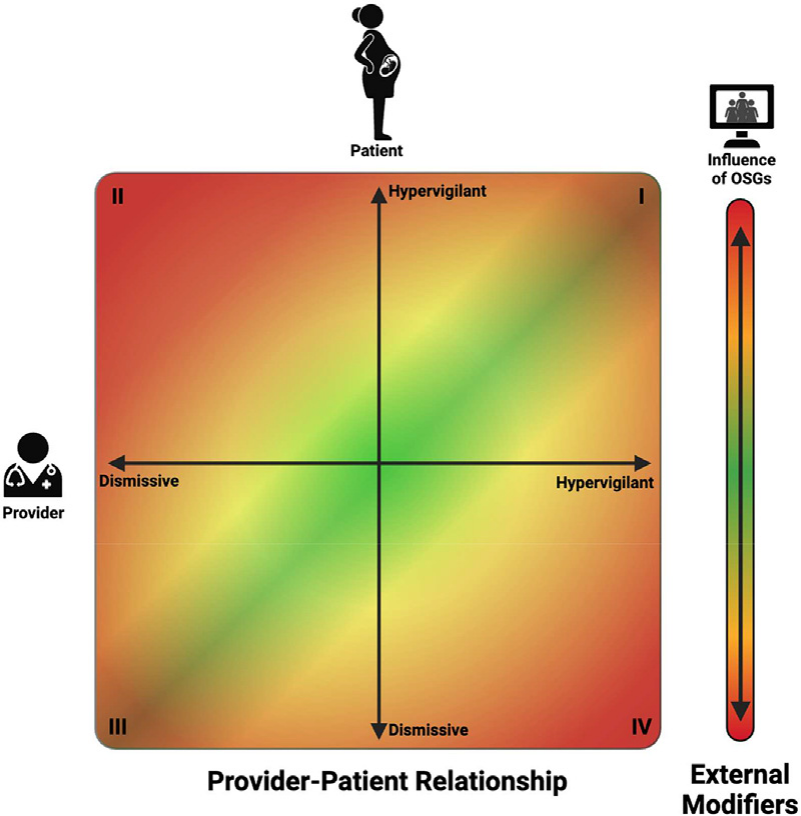
Risk perception congruency model for cardio-obstetric patients and their providers, along with potential external influences The color gradient indicates the patient's feelings of security in their pregnancy and relationship with their care team, with green being most secure and red indicating stress and uncertainty.

We identified 3 major themes that strongly influenced the pregnancy experience: (1) matching of risk perception, (2) importance of communication, and (3) peer influence on risk perception (Figure 1).

Kiley. Antenatal experiences of pregnant women with cardiac conditions. Am J Obstet Gynecol 2025..

### Subtheme 1: Matching of risk perception

**Figure 2:**
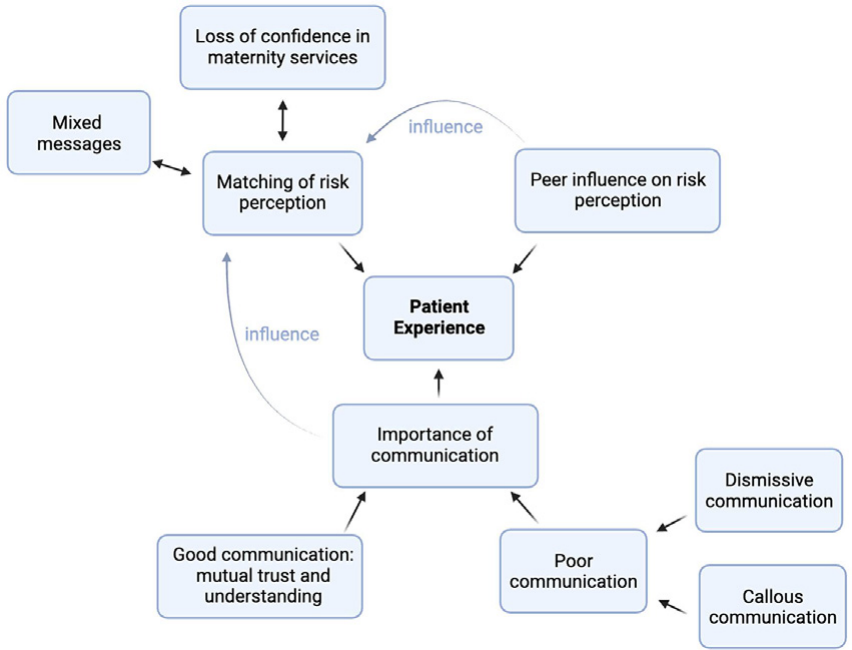
Relationship of themes and their impact on pregnancy experiences of women with a cardiac disease

Women who themselves felt highly anxious about their cardiac condition could still have a positive experience when a care-provider matched this with vigilant monitoring and reassuring communication (Figure 2, Quadrant I). On the other hand, where care-providers were perceived to assess risk as lower than women, this mismatch provoked significant anxiety. When women felt anxious about their cardiac condition, many were reassured by the idea that ‘special care’ was being taken by their providers. Where providers did not perceive the need for this, pregnancy experiences were often confusing or difficult:

*“It was upsetting for me because with my medical past, I believe that I should have been treated a little bit different from a normal person. Because it’s my heart and it is my baby. And they said ‘no’ they wouldn’t.”**^12^* (United Kingdom)

Kiley. Antenatal experiences of pregnant women with cardiac conditions. Am J Obstet Gynecol 2025.

In contrast, other women described the converse situation or were inclined to downplay the severity of their cardiac status (Figure 2, Quadrant IV). This was often associated with feelings of frustration about how their pregnancy was over-managed and over-medicalized (Table 2).

*“that was a huge concern. I don't think that they looked at me as a pregnant woman. I think they looked at me as a cardiac patient”**^10^* (Australia)

(1) Mixed messages

**Table 2:** Examples of women’s quotes on risk perception matching

1. Medicalization	*“I just stayed at home, going to the doctor and undergoing ultrasounds, ultrasounds, ultrasounds, and came here all the time. I thought that I could, because of the job leave, enjoy life, that I would be able to go out with my family, but I can’t do anything, only go to the doctor, go to the doctor, go to the doctor”*^13^ (Brazil)
	*“It’s when you’re very healthy and then you’re told that you’re not healthy … I didn’t feel sick, I didn’t look sick, I didn’t act sick, so it was really challenging for me to comprehend that.”*^11^ (Australia)
	*“[I] was so ‘euphoric’ on conceiving and experiencing [my] pregnancy that [I] needed to be reminded by [my] obstetrician to return for cardiologic review.”*^17^ (Australia)
2. Mixed messages	*“…it was very confusing, I had a lot of mixed messages from various different people telling me completely different things.”*^12^ (United Kingdom)
3. Loss of confidence in maternity services	*“The midwife visits I had, but no one asked about my heart… It’s really sad to be honest because I know how busy all midwives are, but really they’re just doing a box ticking exercise….”*^12^ (United Kingdom)
	*“I go into emergency and some of them have never even heard of what I've got… 1 in 500 people have this, that's really disappointing that some medical professionals have never heard of it, or they don't know how to treat it, or they treat it incorrectly.”*^10^ (Australia)

Women frequently recounted experiences of frustration and confusion when dealing with disjointed care, especially when they felt that their individual circumstances were not known or understood by providers. This was exacerbated in situations where individual providers within a service appeared discordant with 1 another in assessing risk (Table 2).

*“He [the anaesthetist] said that I should have an epidural and caesarean. I said that my cardiologist said that I couldn't have an epidural and he said ‘No, you can’. I said I couldn't and then he left.”*^10^ (Australia)

(2) Loss of confidence in maternity services

Where women felt that providers did not grasp their individual risk status due to lack of familiarity with maternal cardiac conditions, there was often an associated loss of faith in maternity services more generally. Many women felt important aspects of their care could be overlooked. This was especially true when women felt that clinicians were not basing their recommendations on clear evidence or extensive experience (Table 2).

*“The answers I was getting weren't really based on research or on best guidelines or, experience… no‐one could ever really give me real answers, and I felt a bit like that was just their gut feelings.”*^10^ (Australia)

### Subtheme 2: Importance of communication

Women’s experiences of communicating with providers profoundly influenced their chances of reaching a position of concordant risk perception (Figure 2). Many women emphasized that feeling heard by their providers promoted confidence and helped them reach agreement on plans for monitoring and intervention. Women who felt that their providers didn’t hear their perspectives were much less likely to feel they had a positive pregnancy experience or that their needs were met.

(1) Good communication: mutual trust and understanding

For women with pre-existing cardiac conditions, a previous connection with a provider was often associated with feelings of safety, trust, and that there was a shared understanding of risk. While women diagnosed with new cardiac conditions during pregnancy, principally those with PPCM, did not have previous connections to draw on, they also emphasized their relationship with their provider as a key factor in their pregnancy experience. Accurate and timely communication around their diagnosis was often the first building-block of a patient-provider congruence in risk perception, leading to a positive experience (Figure 2, Table 3).

*“My diagnosis was death, my cardiologist was a gift to me in every sense of the word.”*^19^ (OSG)

**Table 3:** Examples of women’s quotes on the importance of communication

1. Good communication	*“[I was] confident with cardiologist and obstetrician as well, felt like I was in good hands”*^18^ (Australia)
	*“I am so blessed to have a wonderful MD, and he is wonderful because he also has heart problems, so he can relate.”*^19^ (OSG)
	*“He [my OB/GYN] listened to my symptoms and my heart, and then sent me directly to the ER. My OB/GYN was the best, I thanked him for saving my life.”*^19^ (OSG)
	*“Sometimes I came here, and we have a lot of lectures here, a lot of support... I virtually lived here in the hospital, and it was my second family”*^13^ (Brazil)
2. Dismissive communication	*“No one believed me… I could not enjoy the pregnancy because I was in stress and worried all the time… difficult to make myself heard…”*^20^ (Sweden)
	*“I had bodily pain, it was painful to breathe and I couldn’t talk… The pain was wandering from 1 shoulder to another and then throughout the body… felt helpless; I had no idea what to do”*^21^ (Sweden)
	*“…even with the breathlessness with me having a known cardiac issue…. I don't remember anyone ever actually just listening to my heart … they're just going to assume that its pregnancy related, not cardiac related.”*^10^ (Australia)
	*“…things started getting crazy… my feet and ankles and calves became so swollen…Again I went to the midwife, but she told me it was ‘normal’”*^20^ (Sweden)
	*“I remember that the ECG didn’t show anything abnormal, so my heart was somehow excluded as the cause of my fatigue, and (the doctors’) ‘explanations’ about my health began again with ‘stress’ and ‘overwhelmed’ etc.”*^9^ (Denmark)
	*“‘Do you feel anxious? You might be having an anxiety attack’ and I was saying to them, ‘No… It's not an anxiety attack’.”*^10^ (Australia)
	*“If they had taken a blood sample, they might have discovered that my heart was affected. I stopped peeing but I didn’t call the nurse-midwife because I had already called so many times and received an answer that there was no reason for worry. There was nothing else I could do but believe in midwives and just wait”*^20^ (Sweden)
	*“My OB and GP, they blew it! I’m still angry at them. I went to them for help and they brushed me off. Thankfully, I had an ER doctor that knew better, and a good cardiologist.”*^19^ (OSG)
	*“The OB actually fussed at me for taking my own blood pressure, which was high. I could have just died at home at any given time. I was so mad. I was in respiratory distress and all they suggest is ‘lay on your left side and drink fluids’... which was drowning me. I had every symptom of CHF.”*^19^ (OSG)
3. Callous Communication	*“The cardiologist came in the next day when no one in my family was there and explained what happened to me and that my heart was functioning at 20%. He told me that I might need a heart transplant and that I was finished being pregnant. Of course, I was hysterical. I called the nurse in, and she could see how upset I was. I think she gave that doctor a talking to, because a few hours later he came and apologized to me.”*^22^ (OSG)
	“I wish that someone had thought about my emotional well-being as much as my physical well-being. Being told [to not have any more children] while laying there; to be in the state that you’re in and have someone throw that at you is SO depressing and saddening. You’re so scared in that moment. It’s not what you need. No one needs to say it. You’re not going to get pregnant…you know… I’m strapped up in an ICU to about 50 cords. I’m not getting pregnant anytime soon. It’s so honestly ridiculous. And I was told by multiple people in the most unkind ways. They walk into my ICU room and are like, ‘You know, you absolutely can never get pregnant again’. It’s just the most inappropriate time to have that discussion.” ^14^ (USA)

Women valued care that felt personalized and attentive. Regular reviews and check-ups contributed to an overall feeling of being secure and well-supported for many women.

*“I felt like the world’s elite team was there for me during labor”*^20^ (Sweden)

(2) Poor communication

By contrast, many women discussed experiences where they had faced difficult communication with their providers, including obstetricians, midwives, and cardiologists. The key themes centered around communication that was perceived as dismissive or callous, and many women explicitly discussed how this communication damaged their trust and affected their risk perception during pregnancy.

### Dismissive communication

Some women who voiced their concerns about symptoms felt dismissed by providers (Figure 2, Quadrants II, III). This was particularly true for women who were diagnosed with new cardiac conditions during pregnancy and thus felt helpless and struggled to feel heard when their symptoms began (Table 3).

*“… Deep down in my heart I knew I was dying, though they do not see me if I die now, difficult to get them to listen. I was looking at the door waiting for someone to come and see how bad I was”**^20^* (Sweden)

A recurrent theme was providers dismissing the physical symptoms of heart conditions as typical for a normal pregnancy. Women had to advocate strongly for themselves to override the providers’ perception of their health status. They felt that many providers tended to attribute physical symptoms to their mental health or emotional stress. Women found this particularly frustrating, contributing to a sense of helplessness and feeling misunderstood. These experiences were most prevalent amongst women who had not yet been diagnosed with a cardiac condition.

*“All these happened because they did not listen to me… They attributed my symptoms as mental and emotional”**^20^* (Sweden)

Exasperation over repeated dismissal of symptoms led to breakdowns in the relationship between women and their providers. Women felt fatigued from constantly explaining their symptoms and eventually stopped trying to voice their concerns. They lost trust and faith in their providers with each experience of dismissal, and the lack of attentive care led to feelings of anger and neglect.

### Callous communication

Women described instances of callous or careless communication from providers, especially those whose long-term prognosis was grave (Table 3).

*“’Your heart is so bad you’ll need a transplant at some point’ … oh, and then he said, ‘Hmmm, that’s if you get a heart.’”**^11^* (Australia)

Many women discussed their experience of being advised against subsequent pregnancy. Women perceived this as a key example of their provider not empathizing appropriately with them, and they felt that providers did not hold these conversations with appropriate levels of nuance and care.

### Subtheme 3: Peer influence on risk perception

Where initial incongruity in the patient-provider understanding of risk was present, the views of other parties could close or widen that gap. Where supportive communication from providers was felt to be lacking, women often turned to experts through lived experience in online communities. Women described seeking the stability and continuity of support that they did not receive in their medical setting (Table 4).

**Table 4:** Examples of women’s quotes about peer influence on risk perception

1. Experts through lived experience	*“Talking to someone who’s the same age who’s done the same kind of things, or is at the same life level with the same conditions, is really helpful.”*^11^ (Australia)
	*“I felt very alone in those early years, and my God it would have helped me just knowing that there were other people. My cardiologist had told me at the time, about a week after my episode he had someone that had the same… I remember thinking, God it would be good to talk to her.”*^11^ (Australia)
2. Feelings of isolation	*“I was told PPCM was very rare, but it’s incredible to read these threads and to not feel alone.”*^19^ (OSG)
3. Finding support lacking in usual networks	*‘‘We are there to listen and understand when our friends and family members may not be able or just cannot grasp what we are feeling.’’*^23^ (OSG)
4. Source of hope	*“I think without those support groups I really would have worried about my mental health. They have absolutely saved me. To be able to … write to people in the middle of the night and say ‘I’m feeling really scared. I can’t sleep. I’m gasping for air’ – obviously I was having anxiety episodes, and people were saying ‘It’s okay. You’ll be okay. This is the same experience I had. This is what it’s all about.”*^11^ (Australia)
5. Advice for others	*“I have thought about the fact that someone might be sitting in bed in a hospital [in the middle of the night], feeling very isolated, and waiting for me to answer and join them to the page, so they could sit there and read.”*^11^ (Australia)
6. Anxiety inducing	*“…at one point I come across a Facebook group. And there I find out that there are alternatives and that there are many people who survive it, but there are also some who die from it. That’s when I first realised that you can die from it. And it frightens me quite a bit”*^9^ (Denmark)
	*“I am kind of glad I didn’t find them early on because you do see some horror stories, and … that there is someone ten years on still experiencing heart attack pain. You don’t want to know that when you’re just out of hospital for the first time.”*^11^ (Australia)

Due to the relatively low incidence of cardiac conditions during pregnancy, peer support was most often accessed in the form of OSGs. A major theme in women’s impetus for joining an OSG was their feelings of isolation upon receiving a diagnosis (Table 4).

*“I really needed to know that there were others out there who were in the same situation. I needed to know that it was OK to be scared and confused. They gave me hope that it wasn’t just a death sentence like the doctors had made it out to be.”**^23^* (OSG)

Women often perceived that their families and ‘real-life’ friends were less able to relate to their experiences during pregnancy with a cardiac condition. They placed high value on hearing the lived experiences of others and on the benefits of mutual support. Many women expressed that connecting with someone with the same diagnosis gave them confidence and made them feel supported in ways that were missing from their usual networks (Table 4).

*‘‘No 1 in this world can truly relate to you like these women. Only they know the pain that is embedded with in my heart because they have all felt it.’’**^23^* (OSG)

In these support groups, women found sources of hope that they lacked elsewhere. OSGs generally helped women feel less anxious about their conditions, and many referenced their online experience as providing valuable hope during difficult pregnancies (Table 4). Women felt compelled to reciprocate within the OSGs and contribute advice to others who shared the diagnosis, which in turn propagated the utility of these resources (Table 4).

By contrast, participating in OSGs could sometimes be anxiety-inducing when women engaged online with other women who did not experience good pregnancy outcomes. This was particularly true among women who felt they had not received enough comprehensive information about their personal condition from healthcare providers and were extrapolating their own prognosis from experiences related on OSGs (Table 4).

## Comment

### Main findings

Understanding how women with cardiac conditions experience pregnancy care is vital to ensure providers and services can meet women’s needs. We found that congruence in patient-provider risk perception strongly influenced pregnancy experience, particularly the confidence women had in the teams caring for them. Whether women and their providers reach a shared understanding of risk is powerfully influenced by the quality of their communication, especially whether women felt adequately heard.

### Comparison with existing literature

We propose a framework to improve understanding of the experience of pregnant women with cardiac conditions (Figure 2). This model provides important context for healthcare providers to understand how their communication style and content can influence a woman’s pregnancy. Our proposed framework augments the findings of a previous systematic review of the experiences of women with cardiac conditions in pregnancy by adding 7 recently published studies and furthering understanding of how effective communication promotes shared patient-provider understanding of risk.^24^ Our review also specifically addresses differences in the antenatal experiences of women with known versus newly diagnosed cardiac conditions.

Patient-provider communication and risk perception matching are closely intertwined.^25^ Women who underestimated their risk relative to their providers largely reported that they felt well during their pregnancy and resented feelings of over-medicalization and unnecessary interference.^26^ This was more common in women who reported that they had not received detailed information about their cardiac condition. Particularly when grappling with the shock of a new cardiac diagnosis, some women were perhaps more receptive to any opportunity for normalization^27^ and to avoid attempts to engage them with close monitoring. We also identified some women with lifelong cardiac conditions who considered themselves to be "normal,"^28^ and had difficulty adjusting to the idea of "additional risk" during pregnancy. When caring for these women, it is important that physicians acknowledge and discuss the increased risks associated with pregnancy within women’s existing frameworks for understanding their condition.

By contrast, women who felt their pregnancy was very high risk often experienced unwanted normalization, engendering feelings of dismissal. Women’s feelings of insecurity and isolation were compounded where they experienced insensitive and callous communication from their providers. Many women who had life-long cardiac conditions had long-standing ideas about what pregnancy would entail and felt unsafe if providers tried to downgrade this assessment.^28^ Conversely, some women with newly-diagnosed cardiac conditions such as PPCM had to battle to have their conditions distinguished from physiological or typical pregnancy symptoms,^29^^,^^30^ often experiencing residual mistrust after a delayed diagnosis. Thus, it is imperative that physicians undertake individualized, informative, and nuanced conversations regarding risk to improve women’s experience during pregnancy.^31^ Focusing on hearing and documenting patient narratives allows care plans to be devised through shared decision-making. Our results suggest that this will augment patient-physician relationships and promote congruence in risk-perception matching.

Our findings also demonstrate some systematic differences between the experiences of women with pre-existing compared to newly diagnosed cardiac conditions during pregnancy. Women with pre-existing cardiac conditions tended to have more confidence in their trusted providers^32^ and fewer experiences of dismissive communication. A focus on relieving patients’ burden of managing and organizing their own complex health histories could further improve these women’s experiences, especially when entering new clinical settings for antenatal care. These efforts may be augmented by robust preconception counseling to prepare for a pregnancy with a cardiac condition. By contrast, women with newly diagnosed cardiac conditions were much more likely to experience feelings of isolation, stress, and uncertainty.^33^ Insecurity and isolation were sometimes compounded by insensitive and callous communication from providers, especially when breaking news about poor prognosis or advising against subsequent pregnancies. It is critical that providers develop an understanding of how their communication in such circumstances is perceived and how it may influence their patient’s ongoing experience during pregnancy.

Lastly, many women seek information, experiences, and insights from multiple sources, including support groups with lived experience, that may strongly influence how they conceptualize their risk of adverse pregnancy outcomes.^34^^,^^35^ Peer interaction can have a strong influence on the evolution of risk perception, sometimes providing a false sense of security and other times contributing to a heightened sense of anxiety.^36^^,^^37^ OSGs are an invaluable resource through which women can access much-needed information and community.^38^ However, the quality of information is variable, which can be detrimental particularly when relying primarily on these groups for answers and advice.

### Strengths and limitations

A strength of this study is the diversity of cardiac diagnoses represented among the women in the included analysis. There was an extensive mix of pre-existing cardiac conditions (either congenital or acquired etiology) and newly diagnosed cardiac conditions. Further specific investigation of these experiences is likely to become possible as the literature in this key area grows. An important limitation of this review is that while nationality or ethnic origin of participants was sometimes reported in the included studies, race and ethnicity were not factors in the ensuing analysis of patient experiences. This was perhaps because the studies which did include these demographics had mainly ethnically homogenous cohorts. Participant demographics were not reported in conjunction with quotes, limiting comparisons across studies. Additionally, while this review encompasses an international data set including 6 countries, most studies came from high-resource settings. Thus, further research is needed to investigate the experiences of cardio-obstetric patients through the lens of potential experiential differences with race/ethnicity and in low-resource areas. There was not sufficient available information in the primary studies for us to assess the impact of maternal education or socio-economic status on pregnancy experience, and this is also an important area for future research.

### Conclusions and implications

Our study has many implications for providers who care for pregnant women with cardiac conditions, including doctors, nurses, and midwives, as well as for the women themselves. It is paramount that providers understand the experiences of these women rather than relying solely on outcome data to inform their clinical practice. Maternal medicine centers that specialize in care for cardio-obstetric patients are of particular importance in ensuring optimal clinical and experiential outcomes for this group. We highlight the importance of nuanced and personalized discussion about the risk of cardiac conditions during pregnancy. Finding a common ground in risk perception is critical to strengthening the patient-provider relationship and providing a basis for women to feel safe and informed during their pregnancy.

## CRediT authorship contribution statement

**Jasmine X. Kiley:** Writing – review & editing, Writing – original draft, Visualization, Project administration, Methodology, Investigation, Formal analysis, Data curation, Conceptualization. **Annabelle Corlett:** Writing – review & editing, Validation, Investigation. **Emma Mitchell-Sparke:** Writing – review & editing, Validation, Methodology. **Brittany Jasper:** Writing – review & editing, Validation, Methodology, Investigation. **Tabitha Wishlade:** Writing – review & editing, Methodology, Data curation. **Catriona Bhagra:** Writing – review & editing. **Sara Wetzler:** Writing – review & editing, Data curation. **Catherine E. Aiken:** Writing – review & editing, Writing – original draft, Validation, Supervision, Methodology, Investigation, Formal analysis, Conceptualization.

## Appendix Supplementary materials

Image, application 1

Image, application 2
