# Supplementary material for: Antenatal experiences of pregnant women with cardiac conditions: a systematic review and meta-synthesis
Source: AJOG Glob Rep. 2025 May 28;5(3):100522. doi: 10.1016/j.xagr.2025.100522 (PMC12221574; doi:10.1016/j.xagr.2025.100522)
Supplement: Supplementary file 1 [file mmc1.pdf]

PRISMA 2009 Flow Diagram

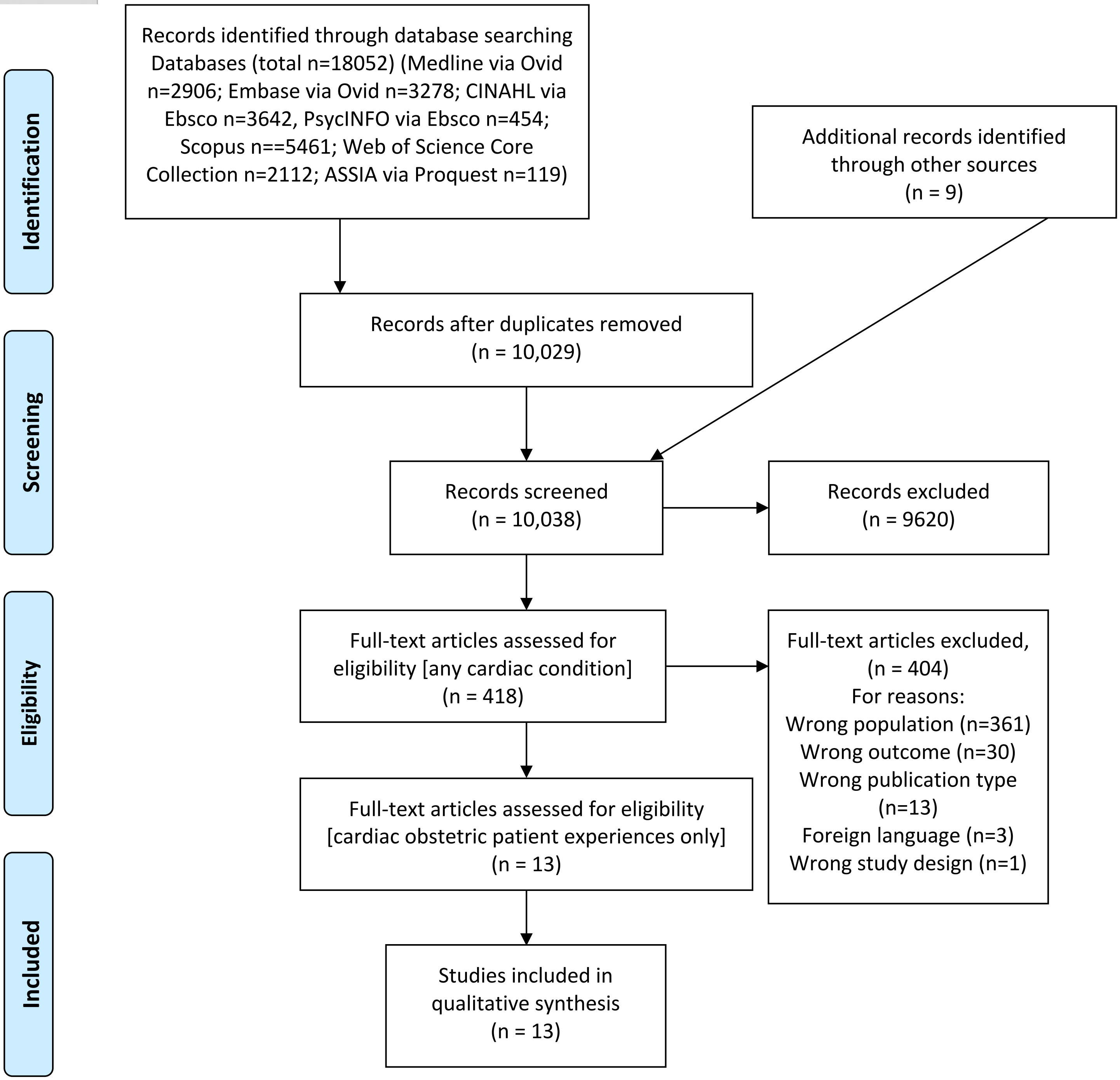

From: Moher D, Liberati A, Tetzlaff J, Altman DG, The PRISMA Group (2009). *P*referred *R*eporting *I*tems for *S*ystematic Reviews and *M*eta-*A*nalyses: The PRISMA Statement. PLoS Med 6(6): e1000097. doi:10.1371/journal.pmed1000097

For more information, visit www.prisma-statement.org.
