# Supplementary material for: Antenatal experiences of pregnant women with cardiac conditions: a systematic review and meta-synthesis
Source: AJOG Glob Rep. 2025 May 28;5(3):100522. doi: 10.1016/j.xagr.2025.100522 (PMC12221574; doi:10.1016/j.xagr.2025.100522)
Supplement: Supplementary file 2 [file mmc2.docx]

**Table A.2:** CASP 2018 evaluation of the included studies.

Review evaluation: 1= study about the experience; 2 = study about the how; 3 = study of the impact of the how’s

| **Review** | **Authors** | **Year** | **Was there a clear statement of the aims of the research** | **Is a qualitative methodology appropriate** | **Was the research design appropriate to address the aims of the research** | **Was the recruitment strategy appropriate to the aims of the research** | **Was the data collected in a way that addressed the research issue** | **Has the relationship between researcher and participants been adequately considered** | **Have ethical issues been taken into consideration** | **Was the data analysis sufficiently rigorous** | **Is there a clear statement of findings** | **How valuable is the research** | **Narrative appraisal** |
| --- | --- | --- | --- | --- | --- | --- | --- | --- | --- | --- | --- | --- | --- |
| 1 | de Wolff, M., Schjodt Ersboll, A., Hegaard, H., Johansen, M., Gustafsson, F., Damm, P., Midtgaard, J. | 2018 | Yes, to explore women’s experiences of the process of regaining psychological balance and wellbeing after having experiences severe peripartum morbidity. | Yes, in-depth, semi-structured, face-to-face, telephone, and email interviews to understand women’s psychological adaptation after having peripartum cardiomyopathy (PPCM). | Yes, interview questions pertained to specified topics such as experiences of motherhood, breastfeeding, and psychological reactions despite being open ended. Probes were specified. | Yes, participants were women with a validated PPCM diagnosis and who could understand and speak Danish. They were recruited from a nationwide population-based study which invited 58 women via letter and phone to the clinic. 28 women accepted, and they were informed about the qualitative study. 14 of them were successfully recruited for interviews. | Yes, face-to-face interviews were conducted in participant homes or in a private room at the research unit and lasted between 28 and 140 minutes. | Not sure, the relationship of the interviewer with the patients and whether they had previously established rapport was not stated. | Yes, approval was obtained from the Danish Data Protection Agency. The Research Ethics Committee of Copenhagen had no objections to the study. All participants gave written informed consent and knew they could withdraw from the study at any time. | Yes, the data was analyzed via thematic analysis. However, only one reviewer coded and performed the analysis, so there was no cross-verification. | Yes, the main findings are clearly described, which fell under the main theme of recovering to a new normal and included losing trust, silence after chaos, disrupted early mothering, choices made for me and not by me, and ability to mobilize inner resources. | This study contributes to limited body of knowledge about psychosocial recovery from PPCM. It sheds light on need for prospective studies that follow PPCM patients and measure their psychological health and impacts of psychological support in the time during and after PPCM. | The findings uncovered the vulnerability of the time after PPCM and difficulty in regaining psychological balance. Also, the prioritization of physical symptoms over mental and emotional ones was palpable during this time. |
| 1 | Dekker, R., Morton, C., Singleton, P., Lyndon, A. | 2016 | Yes, to describe women’s experiences being diagnosed with PPCM. | Yes, publicly available narratives posted by women with PPCM from three online support groups (OSGs) to increase understanding of women’s experiences following PPCM diagnosis. | Yes, data were extracted from postings of women with PPCM diagnoses. | Not sure, there may be bias in the self-selecting nature of those who choose to post on online forums. Additionally, demographic data was extremely limited. | Yes, drawing data from three different OSGs allowed for a slightly more diversified group of study subjects. | Yes, this study was not determined to be human participants research because data was publicly available on the internet. As such, the relationship with participants was not of concern. | Yes, the Stanford University institutional review board determined this study was not considered human-participants research. All postings were anonymized. | Yes, thematic analysis was employed to analyze the data, and multiple investigators participated in study design and data analysis. Additionally, two nurse-researchers, a sociologist, and a social psychologist enhanced analytic triangulation. | Yes, the main findings are clearly described, which included women’s memories of initial dismissal and whether they were taken seriously, feelings of terror and devastation upon diagnosis, struggles with caring for their newborn during postpartum recovery, and struggles with medical advice to avoid subsequent pregnancies. | This study contributes to the small body of work that addresses patient experiences of PPCM and what that looks like in the context of new motherhood and postpartum recovery. It sheds light on the need for studies that look at women’s experiences and assess their emotional and mental health response to diagnosis of PPCM. | The sample size of 92 postings used in this study allowed for numerous perspectives of what the experience of PPCM diagnosis and subsequent time period meant for women. It is clear that many of these women experience symptom dismissal despite the severity of their condition. |
| 1 | Donnenwirth, J., Hess, R.F. | 2018 | Yes, to explore the experiences of women living with PPCM and their decisions regarding a subsequent pregnancy. | Yes, semi-structured interviews allow for investigation into the experience of women with PPCM and what they consider regarding subsequent pregnancies | Yes, the interview questions were guided by modified grounded theory, and sample questions were provided. | Yes, two approaches were used: admission data of a hospital in northeast Ohio and three interviews came from this, and then the remaining participants were recruited from a Facebook PPCM survivor group. | Yes, face-to-face and telephone interviews were conducted. In-person interviews were conducted privately in the hospital or the participants’ homes and lasted about 60 minutes. | Not sure, unclear relationship between researchers and participants and whether they had previously established rapport. | Yes, ethical approval was granted by the Human Research Review Board of the Aultman Health Foundation in Ohio, USA. Women gave oral consent and had the opportunity to ask questions before doing so. | Yes, a modified constant comparison analysis was used. First, both authors conducted the analysis independently before coming together but they determined that data saturation had not been reached. Four more women were interviewed and the data analysis was repeated with adequate data saturation. | Yes, the main findings are clearly described and include receiving the ultimatum of no more children, weighing the risks, making the decision about a subsequent pregnancy, and experiencing a subsequent pregnancy. Nine additional subthemes were explored in the study. | This study adds to the limited body of work pertaining to the process of decision making about a subsequent pregnancy after receiving a PPCM diagnosis. This is highly relevant as PPCM can be so severe and many women struggle with emotional upheaval as they deal with its aftermath. | The psychology behind women’s decision-making about their own health and the future of their family reveals the complex mental state that accompanies a PPCM diagnosis. Evidently, more research is needed to learn about the impact of PPCM on women and on their family members. |
| 1 | Hess, R., Weinland, J. | 2012 | Yes, to describe the contents of postings made on the MySpace PPCM support group website by women diagnosed with PPCM | Yes, the postings from MySpace PPCM support group revealed the concerns, interests, and topics of relevance to women with PPCM who seek an online community. | Yes, having nearly three years’ worth of postings provide a longitudinal look at the journeys of the regular posters in the online community. However, demographic data was extremely limited. | Not sure, there may be bias in the self-selecting nature of those who choose to post on online forums. | Yes, drawing from postings made between September 2005 to May 2008 allowed for thorough exploration of the content discussed in the online community. | Yes, the data was publicly available on the internet so concerns about relationship between researcher and participant were minimized. | Yes, ethical approval was given by the Human Research Committee of a university in the Midwestern United States. Identities of posters were kept confidential. | Yes, the Neuman Systems Model was employed to categorize the themes drawn from the postings. Both authors participated in data analysis, allowing for sufficient cross-checking. | Yes, the main findings were clearly identified and included six themes which encompassed discussion of symptomology, exchange of advice, interactions with healthcare providers, uncertainty about subsequent pregnancies, expression of spiritualist, and recovery from heart failure. | This study adds to the limited body of work investigating the everyday concerns and knowledge of women with PPCM diagnoses. It sheds light on the need for reliable information and counseling on life and future planning with PPCM. | This study provides a useful snapshot of how women found community and shared experiences among those with a relatively rare disease. The large number of postings (247 postings by 156 people) allowed for a diversified view. |
| 2+3 | Hess, R., Weinland, J., Beebe, K. | 2010 | Yes, to explore the determine the benefits of participation in the online support group for peripartum cardiomyopathy based on a survey of active members of the group. | Yes, the descriptive survey containing open-ended and Likert-style questions allowed understanding of the benefits to participating in the online support group and gain both quantifiable and thematic data. | Yes, survey questions were specified and the open-ended questions allowed women to freely express opinions and write as much or as little as they desired. | Yes, the principal investigator joined the online forum and made a posting about the research study. Volunteers reached out via email and received detailed instructions, consent form, survey, and demographic questionnaire, which they then returned via email. There may have been volunteer bias arising from this method of recruitment. | Yes, email communication was sufficient for obtaining the survey responses and collecting sufficient data. | Yes, all contact between researchers and participants were via email. | Yes, ethical approval was received from the Human Research Committee of Malone University in Canton, Ohio. Written confirmation from the moderator of the online forum was obtained stating that the forum was public and that contacting participants was allowed. | Not sure, a thematic analysis appears to have been used to look at the answers to the open-ended survey questions but this and further details are not explicitly stated. | Yes, the main findings were clearly stated, including the central conclusion that OSGs are an important resource for women with PPCM because they allow women to get and share information, exchange stories, be understood by other women, and gain hope. | This study is the first to investigate the benefits of participating in an online community after PPCM diagnosis. The study sheds light on the implications of these findings specifically on the nursing community. | The small convenience sample size of 12 women indicates that this may not be generalizable to a greater population. However, it provides an important foundation for further investigation into the importance of online communities for patients with PPCM. |
| 1+2 | Hutchens, J., Frawley, J., Sullivan, E. | 2022 | Yes, to explore and understand the healthcare experiences of women with cardiac disease in pregnancy and postpartum. | Yes, semi-structured interviews to understand how women with cardiac disease experience healthcare during pregnancy and postpartum. | Yes, an interview guide was used but not provided. | Yes, participants were recruited via Facebook pages and groups/newsletters/email lists of consenting cardiac groups and organization. There may have been volunteer bias arising from this method of recruitment. | Yes, individual phone interviews were conducted and took between 24 and 90 minutes. | Yes, the researchers were all female healthcare professionals with diverse sexual, reproductive, and public health perspectives who are interested in ensuring positive outcomes for women. | Yes, ethical approval was granted by the University of Technology Sydney’s Human Research Ethics Committee. | Yes, an inductive reflexive thematic analysis was employed to analyze data. One team member led the analysis but all team members listened to the interviews and read the transcripts. | Yes, the main findings were clearly stated, which included the 5 themes of struggling to be heard, search for information, research, education and guidelines, care coordination and continuity, and fitting into services designed for other. | This study contributes to the body of work exploring women’s experiences during pregnancy while having a heart condition. In particular, the study identified a need for system improvement through multidisciplinary care, increased clinician knowledge, and patient support. | This study provides a strong argument for the need for more patient centered care for women with cardiac disease and who are pregnant. It contains actionable clinical implications. |
| 1 | Hutchens, J., Frawley, J., Sullivan, E. | 2022 | Yes, to correct the lack of visibility and information on the experiences of women with cardiac disease in pregnancy and the first year postpartum. | Yes, in-depth semi-structured interviews to discover the experiences of women who had acquired, congenital, or genetic cardiac disease during pregnancy or their first year postpartum. | Yes, interview guide used but not provided, although the opening question of “Can you tell me about your experience” was specified. | Yes, women were recruited via social media advertisements and direct and indirect invitations distributed by cardiac support groups between December 2018 to April 2020. Volunteer may have arisen from this recruitment method. | Yes, individual phone interviews were conducted and took between 24 and 90 minutes. | Yes, researchers’ interest in discovering women’s experiences is clear and researchers and participants did not have previously established rapport. | Yes, written or recorded verbal informed consent was obtained from all participants and the University of Technology Sydney’s Human Research Ethics Committee granted ethics approval. | Yes, an interpretive inductive thematic analysis was utilized and all team members listened to and read the interviews. The analysis was informed by critical theory and social constructionism. | Yes, the main findings were clearly stated, including 3 major themes of index events and their emotional and psychological impact, self-perception, identity and worthiness, and isolation and connection. | This study contributes to the limited body of work pertaining to mental health and psychosocial adaptation to pregnancy while having a cardiac disease. It sheds light on the need for investigating specific disease cohorts and long-term outcomes. | This study provides a holistic view of the wide variability and complexity of women’s experiences having a cardiac condition during pregnancy while also drawing recurring themes experienced by women across various demographics. There was a consistent layering of traumatic experiences. |
| 2+3 | Mayer, F., Bick, D., Taylor, C. | 2018 | Yes, to describe the composition and processes of multidisciplinary care between maternity and cardiac services before, during, and after pregnancy for women with cardiac disease, and explore clinicians’ (cardiologists, obstetricians, nurses, midwives) and women’s experiences of delivering/receiving care within these models. | Yes, semi-structured interviews were conducted by an individual with formal training in qualitative research methods and allowed for investigation into the experiences of care that pregnant women with cardiac conditions had. A topic guide was used to steer discussion to experiences in obstetric and cardiac care from pregnancy planning through the late postnatal period. | Yes, the multifaceted approach of case-note audit, interviews, and observation provided a comprehensive view of care coordination of cardiac and obstetric services and patient and clinician experiences within that framework. | Yes, women were sampled from the case-note audit from the two sites of interest. As case-note audit data was anonymized, a member of the care team asked eligible women if they would be willing to speak to the research team. | Yes, face-to-face and telephone interviews took place between April and May 2016. Interviews lasted 20-40 minutes. | Not sure, the relationship of the interviewer with the patients and whether they had previously established rapport was not stated. | Yes, this study was approved by the Nottingham 1 Research Ethics Committee. Written informed consent for interviews and observation were obtained from all participants. | Yes, a thematic analysis was conducted using the Framework method and combined deductive and inductive approach. Three researchers analyzed the interview transcripts in an iterative process. | Yes, the main findings were clearly stated and a key conclusion was that clinician and woman perception of normalcy in pregnancy and birth and its relationship to safe maternity care were often not in agreement. | This study adds to the limited body of work pertaining to how women should receive care from multidisciplinary teams, despite this being recommended by international and gubernatorial guidelines. This study sheds light on the need for evidence-based guidance for integrated care. | This study provides a rich insight into the complexities of multidisciplinary care coordination. There could have been a stronger reliance on the interviews of women patients in this study. Also, the comparison of different sites and multidisciplinary care methods could have been more explicitly stated. |
| 2 | Ngu, K., Hay, M., Menahem, S. | 2014 | Yes, to compare motivations and perceptions of women with moderate to severe congenital heart disease (CHD) to women with low risk CHD who conceived. | Yes, a written questionnaire and semi-structured interviews to understand demographic and symptom information and why women with high risk CHD pursue motherhood despite dangers to themselves and their infant. | Yes, interview questions were specific to the research question and were supplemented by supportive prompts to encourage women to expand on their perceptions, motivations, and understandings of their heart condition. | Not sure, women who were over age 18 and had successfully completed at least one pregnancy were recruited from a tertiary center and private clinics. Women were stratified into a low risk group and high risk group. No further details were provided. | Yes, interviews took place in person or on the telephone and lasted an average of 30 minutes. | Not sure, the relationship of the interviewer with the patients and whether they had previously established rapport was not stated. | Yes, informed consent was obtained from each participant. Ethical approval was granted by relevant hospital and university committees who conform to the 1975 Declaration of Helsinki. | Yes, a thematic analysis was conducted and cross-verification was completed by a second independent review for four of the interviews. | Yes, the main findings were clearly stated and five major themes arose from the interviews, including the influence of external expectations on women to have children, influence of existing relationships from a partner, family, and friends, women’s personal goals and desired life experiences, presumed innate biological urges, termination of pregnancy not being considered a viable option. | This study provides insight into the internal considerations of women who have had high risk pregnancies due to heart disease. A common theme was that women with mild or sever CHD may downplay the severity of their disease in order to rationalize the decision to get pregnant. | This study provides insights into the strength of a woman’s drive for motherhood taking precedence over her own health. It would be useful for them to include more direct quotes from interviews in the article to provide a more personalized persepctive. |
| 2 | Ngu, K., Hay, M., Menahem, S. | 2014 | Yes, to assess the perceptions of women with CHD regarding the severity of their cardiac abnormality and its implications in pregnancy, and whether their motivations w=to conceive were similar to those of women without CHD. | Yes, a semi-structured questionnaire and semi-structured interview allowed understanding of women’s reasons for conceiving and having a pregnancy despite the potential for risk to them or their infant. | Yes, interview questions explored reasons for deciding to conceive, important factors in making that decision, and perception of risk pertaining to their pregnancy, themselves, and their infant. | Yes, CHD patients under the card of cardiologists at Monash Medical Centre in Clayton, Victoria, Australia were recruited. Those without CHD were volunteers who had come to the center for a routine checkup or accompanying a patient. | Yes, face to face or telephone interviews were conducted and they lasted approximately 30 minutes. | Not sure, the relationship of the interviewer with the patients and whether they had previously established rapport was not stated. | Yes, informed consent was obtained from each participant. Ethical approval was granted by Monash Medical Centre and the University of Melbourne. | Yes, a thematic analysis was employed and an independent coder who was not involved with the study reviewed four of the interviews with a moderate level of agreement, and further discussion provided guidance for the development of themes. | Yes, the main findings were clearly stated, including that women are not discouraged from conceiving largely because they downplay the risks associated with their CHD and pregnancy. | This study sheds light on the need for increased physician understanding of CHD and its associated potential health risks in pregnancy so this can be adequately communicated to patients such that they do not downplay the severity of their condition. | While this study provides a clear cut solution, it perhaps neglects to address the other psychosocial influences that subconsciously motivate women to downplay the riskiness of pursuing a pregnancy. It may not be as straightforward as supplying physicians with increased information that they can relay to their patients. |
| 1 | Patel, H., Berg, M., Barasa, A., Begley, C., Schaufelberger, M. | 2016 | Yes, to explore and describe women’s experiences of symptoms in PPCM. | Yes, the interviews allowed exploration of the experiences of PPCM within Swedish medical care specifically relating to their symptoms. | Yes, interview questions pertained to the women’s experiences of PPCM. The opening question and probe examples were provided. | Yes, women were recruited via their medical records from Western Sweden with a diagnosis of PPCM and contacted via telephone. | Yes, face to face or telephone interviews were conducted and lasted between 20 and 90 minutes. In person interviews took place in a private room outside the clinic. | Yes, there was no professional relationship between the first author and the participants. The interviewer established a trusting and confidential relationship with the patients. | Yes, ethical approval was granted by the research ethics committee of Gothenburg. Written consent was obtained by all study participants. | Yes, a qualitative inductive content analysis was used to identify key themes. Three reviewers completed each data phase, followed by discussion for conformity of interpretation. | Yes, the main findings were clearly stated, including the main theme of being caught in a spider web, which comprised the subthemes of invasion of the body by symptoms and a feeling of helplessness. | This study adds to the body of work investigating the experiences of women who have PPCM and the debilitating physical and emotional burden many of them bear. It sheds light on the need for providers to be attuned to the early symptoms of PPCM in order to provide early referral to a specialist. | Early detection of PPCM is paramount to the physical and emotional wellbeing of these women. The study could have slightly more clearly outlined findings before delving into specifics. |
| 1 | Patel, H., Schaufelberger, M., Begley, C., Berg, M. | 2016 | Yes, to explore women’s experiences of health care while being diagnosed with PPCM. | Yes, qualitative interviews to understand the “insider” perspective of healthcare for women with PPCM | Yes, an opening question and probes were provided that delved into the in-depth experiences of women with PPCM. | Yes, women were recruited via their medical records from Western Sweden with a diagnosis of PPCM between 2005-2012 and contacted via telephone. | Yes, face to face or telephone interviews were conducted and lasted between 20 and 90 minutes. In person interviews took place in a private room outside the outpatient clinic. | Yes, the interviewer had no professional relationship with any of the study participants and established a trusting relationship with each woman. | Yes, ethical approval was received from the research ethics committee of University of Gothenburg. Written consent was obtained from all study participants. | Yes, a qualitative content analysis was used to identify key themes. Peer-debriefing ensured confirmability of the results. | Yes, the main findings were clearly specified, with the three subthemes of not being cared about, not being cared for, and not feeling secure culminating to the main theme of exacerbate suffering. | This study adds to the limited knowledge about experience of healthcare for women with PPCM. It sheds light on the need for providers to listen to and act on women’s communication of symptoms of PPCM even if they overlap with normal pregnancy symptoms. | This study is another that highlights the need for increased provider understanding of PPCM. It is crucial for women to be taken seriously when expressing their concerns and symptoms. The findings are consistent with other studies that address similar research questions. |
| 1 | Vasconselos Amorim, T., de Oliveira Souza, I.E., de Oliveira Salimena, A.M., Azevedo Queiroz, A.B., Vasconcelos Moura, M.A., Cardoso de Melo, M.C.S. | 2018 | Yes, to understand the meaning of pregnancy for women with heart disease and to unveil meanings of the worldliness of the being-there-women in reproductive risk in the experience lived/experience of high-risk pregnancy due to heart diseases. | Yes, open-ended interviews with guiding questions about pregnancy allowed women to share their experiences of being pregnant while having a heart condition. | Not sure, guiding questions were provided but it is not clear how these questions were developed. | Yes, women who were treated at a reference hospital for maternal risk in Brazil were recruited. Inclusion criteria included women with a cardiopathy who were experience or had experienced pregnancy, women who underwent prenatal follow-up at the clinic, or who were admitted to that institution. | Yes, the interviews were conducted at a reference facility for maternal risk following telephone contact to explain the purpose of the research and invite the women to participate. The interviews had an average duration of 32 minutes and took place between July-December 2014. | Not sure, it is unclear what, if any, relationship existed between the researchers and study participants aside from the initial telephone contact. | Not sure, the research was conducting according to resolution no. 466/2012 and had approved certificates of presentation for ethical analysis, but no mention was explicitly made about informed consent. | No, Martin Heidegger’s phenomenology and hermaneutical analysis were utilized but discussion was verbose and vague without coming to substantive conclusions. | No, there are vague headings such as knowing the risk and planning and ignoring how to avoid it, and getting surprised when finding out pregnancy; telling how they felt physically and emotionally during pregnancy; feeling safe due to the prenatal follow-up routine without sufficient clarification as to their relevance. | The research sheds light on the importance of intersubjective care that favors health care in their clinical practice. It also highlights weaknesses in the participants’ knowledge of their reproductive risk. | This study has useful primary data but categorizes and analyzes it in a vague manner that detracts from the overall meaning and usefulness for addressing knowledge gaps in the experiences of cardio-obstetric patients. |
